# Spatial-temporal heterogeneity and driving factors of PM_2.5_ in China: A natural and socioeconomic perspective

**DOI:** 10.3389/fpubh.2022.1051116

**Published:** 2022-11-17

**Authors:** Yuanyang She, Qingyan Chen, Shen Ye, Peng Wang, Bobo Wu, Shaoyu Zhang

**Affiliations:** ^1^School of Geography and Environment, Jiangxi Normal University, Nanchang, China; ^2^Key Laboratory of Poyang Lake Wetland and Watershed Research, Ministry of Education, Jiangxi Normal University, Nanchang, China; ^3^Science and Technology College, Jiangxi Normal University, Jiujiang, China

**Keywords:** PM_2.5_, spatial-temporal heterogeneity, natural and socioeconomic factors, driving ability, GTWR

## Abstract

**Background:**

Fine particulate matter (PM_2.5_), one of the major atmospheric pollutants, has a significant impact on human health. However, the determinant power of natural and socioeconomic factors on the spatial-temporal variation of PM_2.5_ pollution is controversial in China.

**Methods:**

In this study, we explored spatial-temporal characteristics and driving factors of PM_2.5_ through 252 prefecture-level cities in China from 2015 to 2019, based on the spatial autocorrelation and geographically and temporally weighted regression model (GTWR).

**Results:**

PM_2.5_ concentrations showed a significant downward trend, with a decline rate of 3.58 μg m^−3^ a^−1^, and a 26.49% decrease in 2019 compared to 2015, Eastern and Central China were the two regions with the highest PM_2.5_ concentrations. The driving force of socioeconomic factors on PM_2.5_ concentrations was slightly higher than that of natural factors. Population density had a positive significant driving effect on PM_2.5_ concentrations, and precipitation was the negative main driving factor. The two main driving factors (population density and precipitation) showed that the driving capability in northern region was stronger than that in southern China. North China and Central China were the regions of largest decline, and the reason for the PM_2.5_ decline might be the transition from a high environmental pollution-based industrial economy to a resource-clean high-tech economy since the implementation the Air Pollution Prevention and Control Action Plan in 2013.

**Conclusion:**

We need to fully consider the coordinated development of population size and local environmental carrying capacity in terms of control of PM_2.5_ concentrations in the future. This research is helpful for policy-makers to understand the distribution characteristics of PM_2.5_ emission and put forward effective policy to alleviate haze pollution.

## 1. Introduction

Rapid urbanization has resulted in serious air pollution, such as haze, dust and other terrible weather frequently, which bring a huge impact on people's lives, industrial and agricultural production (1–3). PM_2.5_ (particulate matter with an aerodynamic diameter ≤2.5 μm), one of the major air pollutants, has been a popular research topic for academics in recent years (4–7). Many studies have shown that prenatal exposure of pregnant women to PM_2.5_ increases the likelihood of respiratory infection and may even cause early-life respiratory tract diseases to newborns (8), and children exposed to polluted air for a long time may increase the possibility of hypertension, asthma, obesity and metabolic disorders (9). Recent researches have also shown that high concentrations of PM_2.5_ are associated with high COVID-19 mortality (10). Thus, PM_2.5_ pollution incidents have affected human and ecological health in the course of rapid socioeconomic development.

Numerous scholars have done extensive research on PM_2.5_ in terms of components and sources, spatial variation and impact factors, and have proposed many corresponding control measures (11–16). Previous studies have shown that natural and socioeconomic factors have a significant impact on PM_2.5_ (2, 17–19). For example, the increase in temperature is conducive to atmospheric flow, which enhances PM_2.5_ dispersion, alike the higher summer temperature (lower heating energy consumption) are beneficial to the reduction of PM_2.5_ concentrations (20). On the contrary, the higher temperatures promote the formation of secondary aerosols from gas precursors, thereby increasing PM_2.5_ concentrations (21). The stronger air movement in areas with high levels of surface fine particles was likely to increase PM_2.5_ concentrations, and air movement also has a diffusion and transport effect on PM_2.5_ concentrations (22, 23), Precipitation and relative humidity played an important role in the deposition of PM_2.5_, and increased relative humidity increases the water-soluble ion content of the air (24).

Meanwhile, some researches have shown that there was an inverted U-shaped relationship between PM_2.5_ concentrations and the economic development in socioeconomic terms (25). Extensive economic development relying on energy and resource consumption will increase pollution sources and cause deterioration of air quality. Yet, the residents will pay attention to environmental conservation and health impacts from pollutants with improving standards of living (26). The Environmental Kuznets Curve (EKC) relationship was observed between per capita GDP and air contaminant (27, 28). Industrial structure is an important indicator of local social and economic development. It is generally believed that secondary industry generally refers to heavy industrial production, which is easy to cause greater environmental pollution. The tertiary industry mainly refers to business, finance, trust and service industries, which are generally considered to have low environmental pollution. The population density has a significant impact on PM_2.5_ emissions across all sectors (3). For instance, the increase in population density led to increased consumption, increased travel and production activities, thereby increasing PM_2.5_ emissions. However, the drivers of natural and social factors on PM_2.5_ at different spatial and temporal scales are not well understood. Therefore, it is necessary to further clarify the effects of natural and social factors on PM_2.5_ drivers at different scales.

China became the world's second-largest economy in 2010 after only 30 years of rapid economic evolution since the reform and opening up, and it has been one of the regions with the highest PM_2.5_ concentrations in the world (13). There had been many studies on the temporal and spatial distribution characteristics and influencing factors of PM_2.5_ in China (29–33). And yet, China is a country with a vast territory, a large population, complex landforms and climate, and unbalanced economic development. It poses major challenges for the research and governance of PM_2.5_. Due to the differences in spatial-temporal scale and methods, the research results of dominant factors for spatial-temporal variation of PM_2.5_ concentrations are different. Many studies indicated that PM_2.5_ concentrations showed a spatial distribution characteristic of high overall in the north and low in the south. The pollution hotspots of PM_2.5_ were mainly concentrated in eastern and central China, especially in the Beijing-Tianjin-Hebei region and its surrounding area. The Chinese government has taken a series of strategies to control air pollution such as the Air Pollution Prevention and Control Action Plan (Action Plan) from 2013. Since the implementation of the Action Plan, PM_2.5_ concentrations have been effectively controlled and have shown a downward trend (34, 35). But there are few studies on the driving forces of the temporal and spatial variation of PM_2.5_ in China since the implementation of the Action Plan using natural and socioeconomic factors.

Therefore, it is necessary to explore the driving of natural and socioeconomic factors on PM_2.5_ concentrations in whole China. The present study focuses on the following questions by collecting the measured data of PM_2.5_, natural and socioeconomic from 252 prefecture-level cities in China during 2015~2019: 1) The ability of natural and socioeconomic factors to drive the spatial distribution of PM_2.5_ concentrations. 2) Whether natural or social factors dominate the main causes of PM_2.5_ concentrations changes over time at different spatial scales. The result of this study may be useful to the government in the prevention and control of PM_2.5_ concentrations in industrial restructuring and population development planning.

## 2. Materials and methods

### 2.1. Study area and data source

The data in this study was primarily divided into PM_2.5_ data from 252 prefecture-level cities in China from 2015 to 2019, as well as related to urban natural and socioeconomic factors. PM_2.5_ data were obtained from hourly PM_2.5_ monitoring data by the China National Environmental Monitoring Centre (CNEMC, http://www.cnemc.cn/). Hourly PM_2.5_ data were compiled into the daily average data of 252 cities according to the China Ambient Air Quality Standards (GB3095-2012), the China Ambient Air Quality Assessment Technical Regulations (HJ663-2013) and other relevant regulations. Cities with multiple monitoring stations had their data averaged and treated as daily urban average data. Based on previous studies and the physical geographic features of the country, 252 cities in the country were divided into seven major zones (Supplementary Figure S1 and Supplementary Table S1).

According to previous research (17, 20, 30, 36), eight main influencing factors were finally selected from the twelve factors preliminarily screened by the collinearity treatment. Natural factors include relative humidity (RH), temperature (TEMP), wind speed (WS) and precipitation (PCP), which were taken from the National Weather Science Data Centre (NWSDC). The annual-average values of RH, TEMP and WS were obtained on the average daily data. PCP was the total annual precipitation of the city. The secondary industry was selected as the driving factor of industrial structure on PM_2.5_ in the majority of previous research. With the increase of the proportion of tertiary industry, its impact on PM_2.5_ concentrations needs to be studied. Per capita GDP indicators generally represent the level of local economic development. So the socioeconomic factors included per capita GDP (GDPP), secondary industry share (SI), tertiary industry share (TI) and population density (PD) (year-end total population/total area of jurisdiction) from the China Urban Statistical Yearbook, with the missing data was interpolated by contemporaneous neighboring or around areas. The statistical description and overall spatial distribution of the eight selected driving factors from 2015 to 2019 are showed in Table 1 and Supplementary Figures S2, S3.

**Table 1:** Description of the data used in this study.

**Indicators**	**Data source**	**Symbol**	**Unit**	**Mean**	**SD**	**Minimum**	**Maximum**
Relative humidity	Resource and	RH	%	68.74	10.74	29.81	99.61
Temperature	Environmental	TEMP	°C	14.43	5.31	–0.20	25.44
Wind speed	Science data	WS	m/s	2.21	0.67	0.74	6.44
Precipitation	Center	PCP	mm	1034.85	602.39	21.45	4102.50
Per capita GDP		GDPP	CNY	69110.59	36309.00	15356.00	217313.00
Secondary industry share	China City	SI	%	42.41	10.08	10.68	72.90
Tertiary Industry share	Statistical	TI	%	48.12	9.67	26.12	83.52
Population density	Yearbook	PD	person/*km*^2^	408.73	347.95	1.66	2836.22

### 2.2. Study methods

#### 2.2.1. Spatial autocorrelation analysis

Spatial autocorrelation analysis is a model to explore the similarity or correlation of spatial proximity observation results. Global spatial autocorrelation analysis focused on the correlation between observations in close proximity (37). Global Moran's *I* is the most widely known and used statistic to test for the presence of spatial dependence in observations. The Moran's *I* can be calculated using Eq:


(1) $$\begin{array}{l} I=\frac{\sum_{i-1}^{n}\sum_{j-1}^{n}w_{ij}\left(x_{i}-\bar{x}\right)\left(x_{j}-\bar{x}\right)}{\frac{1}{n}\sum_{i-1}^{n}{\left(x_{i}-\bar{x}\right)}^{2}*\sum_{i-1}^{n}\sum_{j-1}^{n}w_{ij}} \end{array}$$

where $\bar{x}=\frac{1}{n}\sum_{i-1}^{n}x_{i}$, *n* is the number of spatial units (in this study, *n* =252); *x*_*j*_ and *x*_*j*_ are the observations of spatial units *i* and *j*, respectively; *w*_*ij*_ is an element of the spatial weight matrix *W* which describes the spatial arrangement of all the spatial units in the sample, where *w*_*ij*_ = 1 if spatial units i and j share a common border and *w*_*ij*_ = 0 otherwise. Values of Global Moran's *I* range from –1 to 1; a positive (or negative) correlation exists among the observations if 0 <*I* <1(or -1 <*I* <1), and the observations are distributed randomly (no correlation) in the space if *I* is close to or equals 0.

The significance of Global Moran's *I* is commonly measured by the standardized statistic *Z* as shown in Eq:


(2) $$\begin{array}{l} Z\left(I\right)=\frac{I-E\left(I\right)}{\sqrt{Var\left(I\right)}} \end{array}$$

where *E*(*I*)and *Var*(*I*) are the expected value and variance of Moran's *I*, respectively; the methods used to calculate them are listed in the Supplementary materials.

The specific location and distribution pattern of local spatial clustering were further determined by a local spatial autocorrelation. The local Moran's *I* was represented by local indicators of spatial association (LISA), which were calculated as follows:


(3) $$\begin{array}{l} LISA=\frac{\left(x_{i}-\bar{x}\right)}{\sqrt{S^{2}}}\underset{j}{\sum }w_{ij}\left(x_{i}-\bar{x}\right) \end{array}$$

(4) $$\begin{array}{l} S^{2}=\frac{1}{n}\overset{n}{\underset{i=1}{\sum }}\left(x_{i}-\bar{x}\right) \end{array}$$

A local spatial autocorrelation analysis can detect four cluster types with statistical significance: high-high clusters (high-incidence areas enclosed by high incidence areas); high-low clusters (high incidence areas enclosed by low-incidence areas); low-high clusters (low-incidence areas enclosed by high-incidence areas); and low-low clusters (low-incidence areas enclosed by low-incidence areas). The results were visualized in ArcGIS 10.6 software.

#### 2.2.2. Geographically and temporally weighted regression model

The geographically and temporally weighted regression (GTWR) model (38) can effectively deal with Spatial-temporal non-stationarity by introducing a temporal dimension based on spatial heterogeneity. This model can simulate PM_2.5_ concentrations at a higher spatial resolution and accuracy across China than some previous models (39). The basic formula is as follows (40):


(5) $$\begin{array}{l} Y_{i}=\beta_{0}\left(\mu_{i},\nu_{i},t_{i}\right)+\sum \beta_{k}\left(\mu_{i},\nu_{i},t_{i}\right)X_{i}t+\varepsilon_{i} \end{array}$$

where (μ_*i*_, ν_*i*_, *t*_*i*_) is the spatial-temporal coordinate of the *i*th sample; μ_*i*_, ν_*i*_, *t*_*i*_ are the longitude, latitude and time of the ith sample point, respectively; β_0_(μ_*i*_, ν_*i*_, *t*_*i*_) denotes the regression constant at the *i*th sample point, i.e., the constant term in the model; *X*_*it*_ is the value of the *k*th independent variable at the *i*th point; ε_*i*_ is the residual; β_*k*_(μ_*i*_, ν_*i*_, *t*_*i*_) is the kth regression parameter for the *i*th sample point, which is estimated as follows:


(6) $$\begin{array}{l} \hat{\beta }\left(\mu_{i},\nu_{i},t_{i}\right)={\left[X^{t}W\left(\mu_{i},\nu_{i},t_{i}\right)X\right]}^{-1}X^{T}W\left(\mu_{i},\nu_{i},t_{i}\right)Y \end{array}$$

where $\hat{\beta }\left(\mu_{i},\nu_{i},t_{i}\right)$ is the estimated value of β_*k*_(μ_*i*_, ν_*i*_, *t*_*i*_); *X* is the matrix of independent variables; *X*_*t*_ is the transpose of the matrix; *Y* is the matrix of composition in the sample; *W*(μ_*i*_, ν_*i*_, *t*_*i*_) is the spatial-temporal weight matrix. *W* is chosen as the Gaussian distance function, the spatial-temporal weight matrix is obtained using the bi-square spatial weight function, and the spatial-temporal distance between sample *i* and sample *j* is:


(7) $$\begin{array}{l} d_{ij}=\sqrt{\delta \left[{\left(U_{i}-\mu_{j}\right)}^{2}+{\left(v_{i}-\mu_{j}\right)}^{2}+\mu {\left(t_{i}-t_{j}\right)}^{2}\right]} \end{array}$$

where the choice of bandwidth affects the establishment of spatial-temporal weights, this paper adopts the Akaike Information Criterion (AICc) law for adaptive bandwidth.

#### 2.2.3. Stability estimation of coefficients

To analyze the spatiotemporal heterogeneity of each variable, we applied the Kernel function to check the stability of the correlation coefficients, and use the coefficient distribution to observe the spatiotemporal characteristics (41). The density function of the variable *x* is as follows:


(8) $$\begin{array}{l} f\left(x\right)=\frac{1}{Nh}\overset{n}{\underset{i-1}{\sum }}k\left(\frac{x_{i}-\bar{x}}{h}\right) \end{array}$$

where *x*_*i*_ is the coefficients subordinated to independent and identical distributions. $n,h,\bar{x}$ represent the number of *x*, bandwidth and mean value, respectively. The Epanechnikov function was adopted as the kernel function for estimation in this work.

## 3. Results and analysis

### 3.1. Spatial-temporal characteristics of PM_2.5_ concentrations

**Figure 1:**
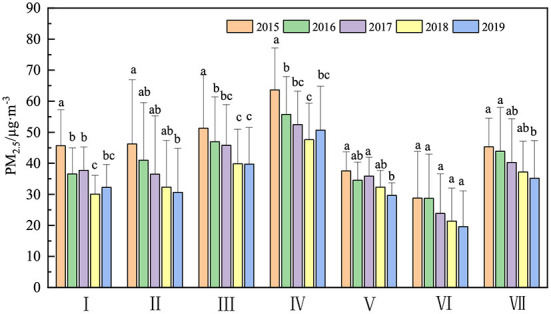
Variation characteristics of PM_2.5_ in different regions from 2015 to 2019 (I) Northeast China, (II) North China, (III) East China, (IV) Central China, (V) South China, (VI) Southwest China, and (VII) Northwest China (Letters denote the result of One-way ANOVA).

**Figure 2:**
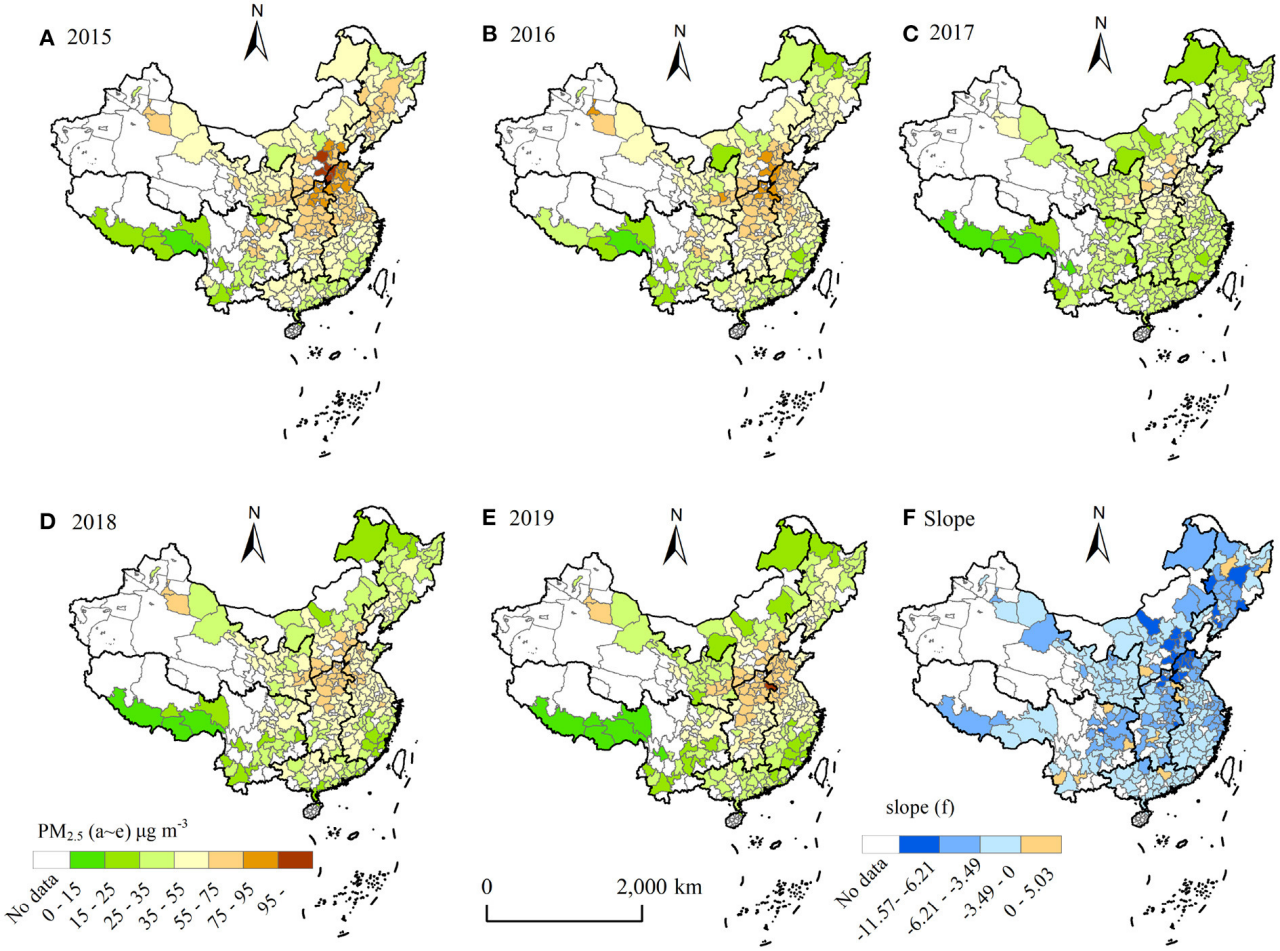
Spatial variation characteristics of PM_2.5_ concentrations during 2015-2019 [**(A–E)** denote the spatial distribution of PM_2.5_ concentrations in 2015, 2016, 2017, 2018, and 2019, respectively, and **(F)** denotes the slope of PM_2.5_ concentrations from 2015 to 2019, slope (5-year linear trend slope)].

The Central, East, Northwest and North China were the regions with high mean PM_2.5_ concentrations, which were 54.03 ± 13.86, 44.71 ± 14.52, 40.38 ± 12.55, and 37.34 ± 18.53 μg m^−3^, respectively, (Figures 1, 2). The mean concentrations of PM_2.5_ in southwest China was lowest (26.50 ± 13.41 μg m^−3^), followed by South China (33.98 ± 5.95 μg m^−3^). The area-weighted mean concentrations of PM_2.5_ in China from 2015 to 2019 were 44.24 ± 17.68, 40.24 ± 15.76, 37.54 ± 14.66, 33.19 ± 12.60, 32.52 ± 13.77 μg m^−3^, respectively, and it was 37.55 ± 15.62 μg m^−3^ in 5 years. The mean concentrations of PM_2.5_ exceeded the annual PM_2.5_ grade II standard (35 μg m^−3^) (GB3095 2012) in 2015, 2016 and 2017, which exceeded 26.4 % in 2015, and 5.2%, 7.1% below annual PM_2.5_ grade II standard in 2018,2019, respectively. The downward trend of PM_2.5_ concentrations was 3.58 μg m^−3^ from 2015 to 2019, with a percentage decrease of 26.49% in 2019 compared to 2015. PM_2.5_ concentrations of 240 cities showed a decreasing trend among the 252 cities, with the proportion of decreasing cities reaching 95.24%, of which 42 cities had a decrease rate of more than 5 μg m^−3^ a^−1^, accounting for 16.67% of the total number of decreasing cities (Figures 1, 2). Although whole regions presented a downward trend from 2015 to 2019, PM_2.5_ concentrations exhibited an obvious spatial heterogeneity in the different regions. The largest decline occurred in North China (–3.99 μg m^−3^ a^−1^), followed by central China (–3.41 μg m^−3^ a^−1^). The region with the smallest decline was North China (-1.80 μg m^−3^ a^−1^). In this study, the slope of change was divided into four classes according to the natural breakpoint method [strong negative (-11.5~ -6.21 μg m^−3^ a^−1^), mid negative (-6.21~ -3.49 μg m^−3^ a^−1^), weak negative (–3.49 μg m^−3^ a^−1^) and weak positive (0~5.03 μg m^−3^ a^−1^)]. It can be found that the strong negative area was mainly located in the Beijing-Tianjin-Hebei region and parts of the northeast, the mid negative region was mainly located in Central China and Cheng-Yu Region, East China, Northeast China and the majority of the other areas were weak negative growth regions. The weak positive area was scattered throughout the country without obvious aggregation areas (Figure 2F).

To further detect local agglomeration of PM_2.5_ concentrations, we adopted a local Moran's *I* test (Table 2). From 2015 to 2019, the average value of the global Moran's *I* was 0.57(*p* <0.01), indicating that PM_2.5_ concentrations showed a club convergence trend. In addition, we also calculated local Moran's *I*, the results of which revealed a detailed local pattern of spatial clustering with changes in PM_2.5_ concentrations. The Moran's *I* value showed a trend of decreasing and then increasing, with the lowest value in 2017 and the highest values in 2018 and 2019, which indicated that an overall trend toward aggregation.

**Table 2:** Global spatial autocorrelation test.

**Year**	**Moran'I**	**Z**	***P***
2015	0.57	26.55	0.001
2016	0.56	26.17	0.001
2017	0.52	22.83	0.001
2018	0.59	27.26	0.001
2019	0.59	26.35	0.001

**Figure 3:**
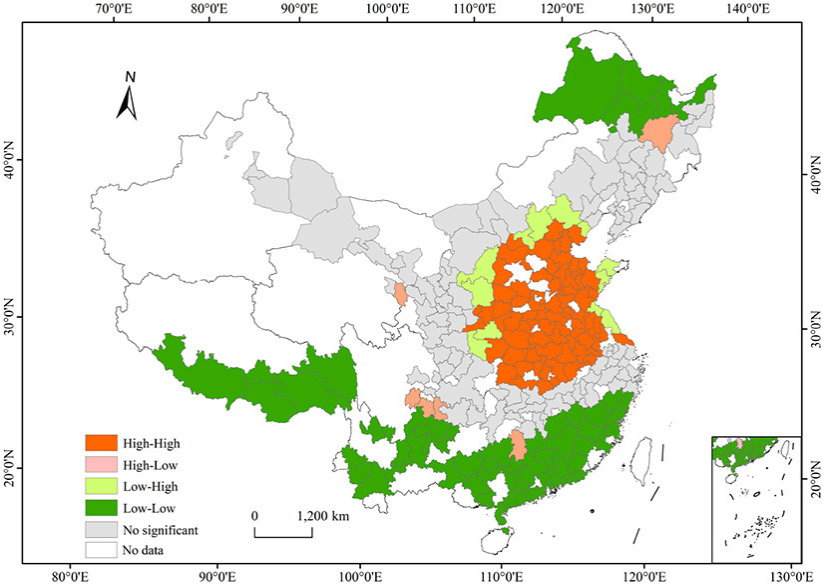
Average of spatial clustering for PM_2.5_ concentrations from 2015 to 2019.

It was discovered that high-high clusters regions were primarily distributed in China's East-central region, including Beijing, Tianjin, Hebei, Shaanxi, Shanxi, Henan, Hubei, Anhui, and Shandong provinces through local spatial autocorrelation analysis. In contrast, low-low clusters were mainly located in the south and southwest provinces of China, including Tibet, Sichuan, Yunan, Guangxi, Hainan, Guangdong, and Fujian. In addition, low and low aggregation areas also appeared in northeastern Inner Mongolia and northwestern Heilongjiang. The high-low agglomeration area and the low-high agglomeration area were small in scope and are distributed near the high-high and low-low agglomeration areas (Figure 3).

### 3.2. Driving forces of variation of PM_2.5_ concentrations

This study selected the GTWR model to analyze the driving forces of temporal and spatial variation of PM_2.5_ concentrations. In order to avoid the deviation of the estimation results caused by the interaction between the indicators, eight driving factors were determined by collinearity test. The results showed that the variance inflation index of each factor was less than 10, and the condition index was also less than 30, indicating that the factor selected in this study does not have a collinear relationship (Table 3). At the same time, in order to avoid the influence of data on the magnitude, PM_2.5_ concentrations and eight driving factors were standardized before modeling. Then the temporal and spatial non-stationary relationships were modeled using the plug-in for ArcGIS 10.6 (with automatic optimal bandwidth settings) in GTWR produced by Huang et al. (38). The AICc value of the GTWR model was -2736.53. The determination coefficient (*R*^2^) and adjustment determination coefficient ($R_{adj}^{2}$) of the GTWR model were 0.78. To evaluate the validity of GTWR results, ordinary least squares regression (OLS) was chosen to compare with geographically weighted regression (GWR), which describes the relationship between variables by building a global model, while GWR expresses the spatial non-stationarity of the relationship between variables through a local model with spatial dependence of parameters. The results showed that AICc values of the GTWR model were lower than those of the OLS and GWR models, and the *R*^2^ was significantly higher, indicating that GTWR results were better than those of the OLS and GWR models (Table 4). The GTWR model coefficients can reflect the direction and intensity of PM_2.5_ driving capability. The positive value indicates the positive driving effect of explanatory variables on PM_2.5_ concentrations, and higher values indicate higher drive capacity, while negative coefficients indicate the opposite.

**Table 3:** Co-linearity test and coefficients statistic description of variables.

**Factors**	**Co-linearity test**			**GTWR coefficients statistic description**				
	**Standardization coefficient**	**Tolerances**	**VIF**	**Median**	**Mean**	**SD**	**Minimum**	**Maximum**
Intercept	-	-	-	0.38	0.42	0.23	–0.22	1.43
RH	–0.04	0.60	1.68	–0.02	0.08	0.10	–0.44	0.29
TEMP	–0.01	0.31	3.22	0.00	0.26	0.31	–0.69	0.81
WS	–0.10	0.80	1.26	–0.11	0.13	0.12	–0.44	0.27
PCP	–0.30	0.41	2.44	–0.42	0.43	0.34	-3.13	0.22
GDPP	–0.19	0.66	1.51	–0.09	0.11	0.11	–0.29	0.49
SI	0.34	0.34	2.94	0.19	0.22	0.21	-1.47	0.62
TI	0.14	0.36	2.77	0.01	0.11	0.16	–0.91	0.47
PD	0.39	0.66	1.52	0.44	0.55	0.74	0.00	8.65

The mean value is the average of the absolute values of the coefficients.

**Table 4:** Result of accuracy evaluation of different model.

**Model**	**AICc**	***R* ^2^**	** $R_{adj}^{2}$ **
OLS	–1606.03	0.34	–
GWR	–2501.28	0.71	0.70
GTWR	–2736.53	0.78	0.78

### 3.3. Stability analyzes of coefficients

**Figure 4:**
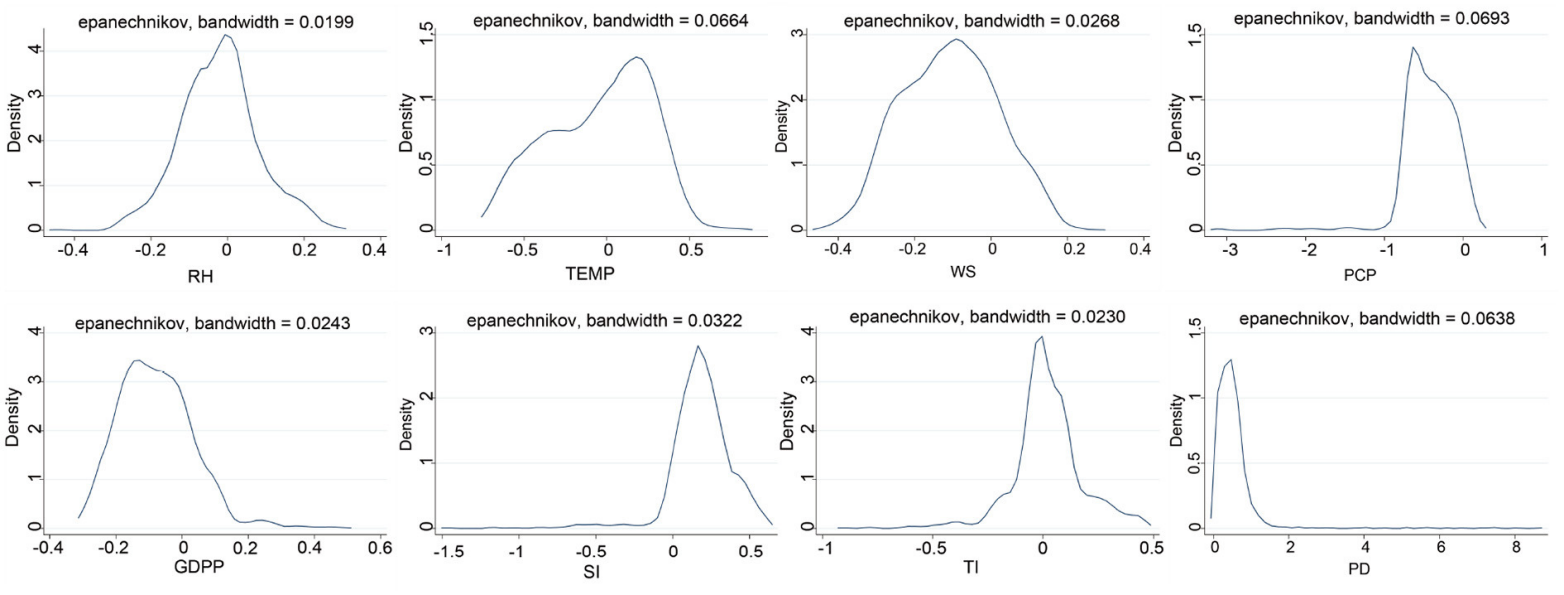
Kernel density distribution of each variable coefficient.

From the Kernel distribution of coefficients of different variables (Figure 4), we can see that the coefficients of RH, TEMP, WS and PCP in natural factors were concentrated at approximately –0.01, 0.2, –0.1, and –0.7, respectively. This result indicates that the increase in WS and PCP had a opposite effect on PM_2.5_ concentrations in most cities, while the increase in TEMP had the promotion effect. Among the four socioeconomic factors we analyzed, the largest density of coefficients of PD was distributed at 0.5 (almost no negative values), which illustrates that with the increase in PD, PM_2.5_ concentrations in most cities were promoted. Simultaneously, the coefficients of SI was distributed at 0.2, indicating that the increase in SI will increase PM_2.5_ concentrations of most cities. In contrast, the coefficients of GDPP was left-distributed, and the peaks emerged at approximately –0.16, indicating that the increase of GDPP is beneficial to reduce the urban PM_2.5_ index in most cities during our study period.

### 3.4. Spatial distribution characteristics of the factor driving force

**Figure 5:**
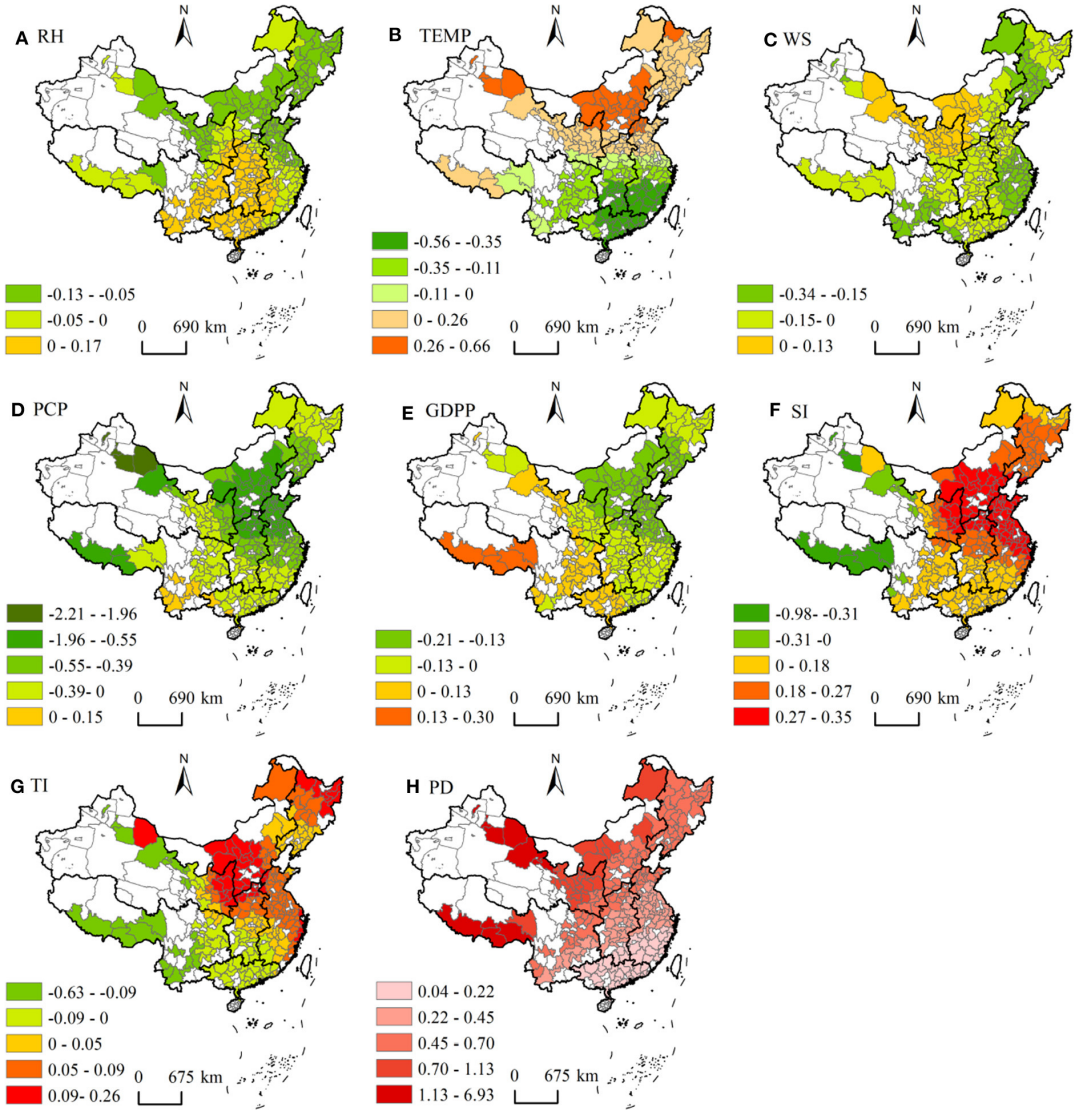
Spatial distribution of coefficients for each driving factor **(A)** Relative humidity, **(B)** Temperature, **(C)** Wind speed, **(D)** Precipitation, **(E)** per capita GDP, **(F)** Secondary industry share, **(G)** Tertiary Industry share, and **(H)** Population density; The white area represents no data.

The GTWR model demonstrated that the force of the driving factor presented different driving distribution characteristics in China (Figure 5). The coefficients of natural factors on PM_2.5_ concentrations were bidirectional at the national scale. RH, WS and PCP showed predominantly negative correlations. Through the analysis of the absolute value of the coefficient, influence intensity of natural factors on the regional PM_2.5_ concentrations was as follows: PCP (0.43) >TEMP (0.26) >WS (0.13) >RH (0.08). The RH coefficient was between –0.13 and 0.17. The proportion of cities with negative driving factors accounts for about 66.67% of all cities. The core region with the strongest negative impact of RH was the northeast, northwest and north China, while central China, southwest and southern China were dominated by weak positive regression coefficients. The TEMP coefficient showed positive and negative equivalence (–0.56 ~ 0.66), and positive correlation regions (48.41%) were mainly distributed in North China, Northeast China and Northwest China. The WS coefficient was mainly negative, accounting for 86.11%, and it was mainly located in the eastern, northeastern and southwestern, and the positive effect was mainly in the northwest region. The PCP coefficient of most cities (95.24%) was negative, and the high negative value area was mainly distributed in northwest and north China.

In socioeconomic factors, except that PD was positively correlated with the PM_2.5_ concentrations in each region, other factors presented a two-way impact on PM_2.5_ concentrations. The order of the absolute values of the driving factors for PM_2.5_ was PD (0.55) >SI (0.22) >GDPP (0.11) >TI (0.11). The coefficient of GDPP has a positive effect on PM_2.5_ concentrations in Southwest and Northwest China (21.03%), while North, Northeast and East China showed a negative driving relationship. The SI coefficient was mainly positive (94.05% of the total number of cities), which was negatively correlated only in the underdeveloped western region, while positively correlated in the central and eastern regions. The TI coefficient range from –0.63 to 0.26. Positively driven cities (67.46% of the total) were mainly distributed in North, Northeast and East China, but the number of cities is significantly lower than that of SI (94.05%). In particular, PD coefficient was positive throughout the region and vary considerably (0.04 to 6.93).There was an increasing trend from southeast to northwest. The lowest region was located in Guangdong and Fujian, the highest region was distributed Tibet, Inner Mongolia, Gansu and Xinjiang (Figure 5).

### 3.5. Temporal characteristics of driving factors

**Figure 6:**
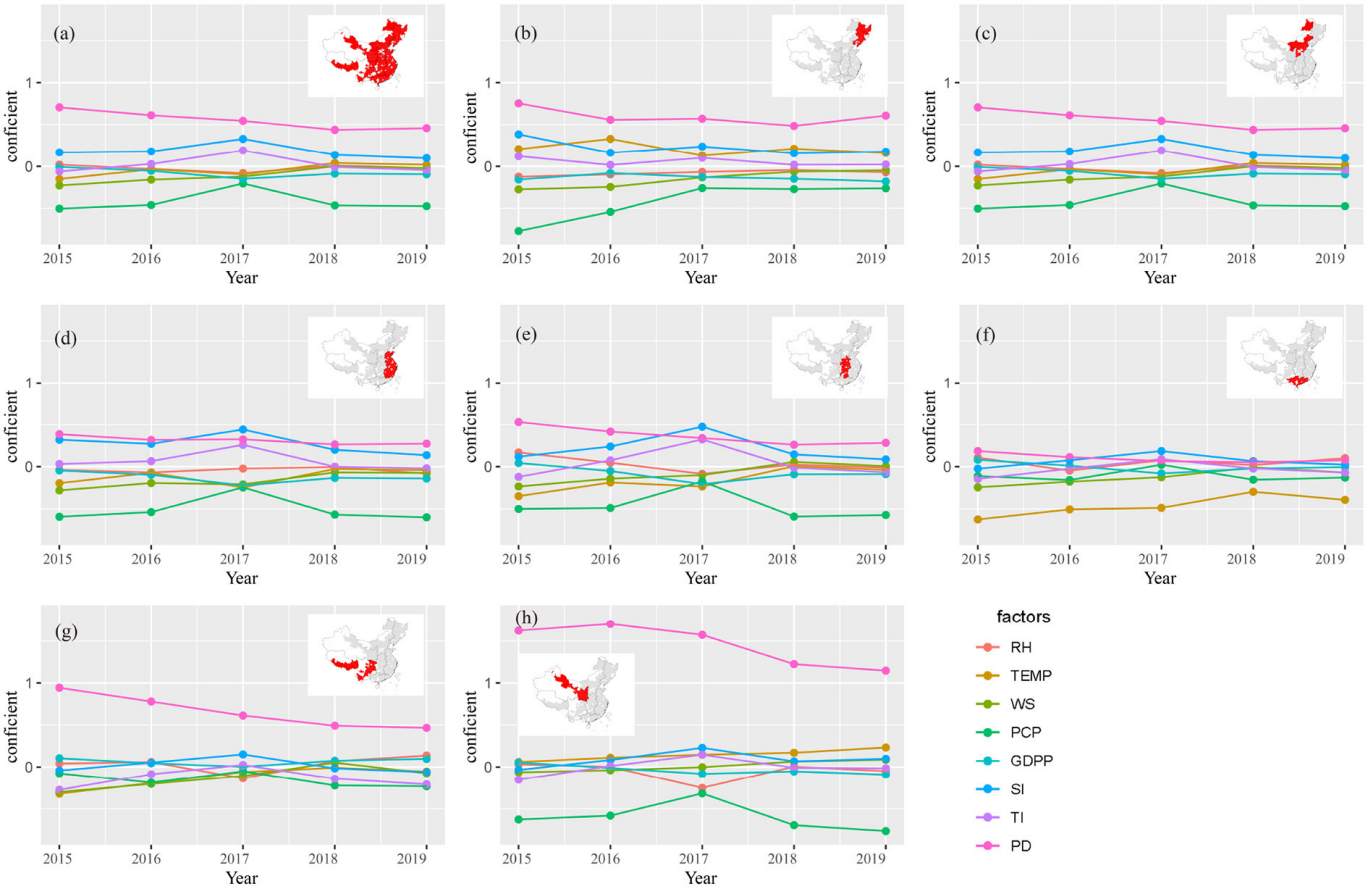
Trends in driving capability of each factor in different spaces from 2015 to 2019 **(a)** Whole region, **(b)** Northeast China, **(c)** North China, **(d)** East China, **(e)** Central China, **(f)** South China, **(g)** Southwest China, and **(h)** Northwest China.

The result of the GTWR model demonstrated that the capability of driving factor was different in time scale (Figure 6). The coefficient value of the eight driving factors was between –0.50 and 0.71 in the whole region. PD (0.55) was highest average positive driving factor, followed by SI. The highest negative driving factor was PCP (–0.42), followed by WS, and the absolute values of the average coefficient of the other factors were all less than 0.05. From the analysis of the time trend, PD has the obvious downward trend (*slope* = 0.07), WS has the significantly upward trend (*slope* = 0.06), and the trends in other factors were not significant (|*slope*| ≤ 0.04).

From 2015 to 2019, the coefficient ranges of driving factors in Northeast and North China were –0.77 ~ 0.75 and –0.76 ~ 0.91, respectively (Figure 6). Positive driving factors of highest average value were PD (0.60, 0.67) in these two regions, followed by SI (0.22, 0.29) and TEMP (0.21, 0.32). The highest negative factor was PCP (–0.42, –0.76) in these two regions. Negative driving factors included WS (–0.15), GDPP (–0.14) and RH (–0.08) in Northeast China and included GDPP (–0.18) and RH (–0.07) in North China, the slope of other factors tend to be stable. From the analysis of the time change trend, the coefficient of PD and SI has obvious downward trend in Northeast China (*slope* = –0.04), PCP and WS has an upward trend (*slope* = 0.13, 0.06), and the annual trend of RH, TEMP, GDPP and SI were no obvious (|*slope*| ≤ 0.02). In North China, PD and SI had obvious downward trend (both *slope* = –0.04), PCP and WS had an upward trend (*slope* = 0.13, 0.06), the annual trend of RH, TEMP, GDPP, SI were no obvious (|*slope*| ≤ 0.02).

The coefficient ranges of driving factors in East, Central and South China were –0.60 ~ 0.44, –0.59 ~ 0.53 and –0.63 ~ 0.19 from 2015 to 2019 (Figure 6). The driving factor of the highest positive coefficient was PD (0.31, 0.37, 0.10) in the three regions, followed by SI. PCP (–0.51, –0.47) was the driving factor with the highest negative coefficient in East and Central China. The driving factor of the highest negative coefficient was TEMP (–0.46) in South China. The coefficients of SI and PD decreased significantly from 2015 to 2019 (*slope* = –0.04, –0.03) in East China. The coefficients of PD in Central and South China had a relatively obvious downward trend (*slope* = –0.06, –0.03). The coefficients of WS and TEMP had an obvious upward trend in East, Central and South China. The change trends of the other driving factors were no obvious (|*slope*| ≤ 0.03).

The coefficient ranges of driving factors were –0.32 ~ 0.94 and –0.77~1.70 in the southwest and northwest regions from 2015 to 2019 (Figures 6G,H and Supplementary Figure S2). The driving factors of highest average positive coefficient were both PD (0.66, 1.4) in these regions, followed by GDPP (0.06) and TEMP (0.14,) respectively. The driving factor of the highest negative was PCP (–0.15,–0.60) in both the southwest and northwest, while other driving factors were no obvious (|*slope*| ≤ 0.08). The coefficient of PD decreased significantly in the southwest and northwest from 2015 to 2019 (*slope* = –0.12, –0.14). The coefficients of TEMP and WS in the southwest region had an upward trend (*slope* = 0.07). The trends of the other driving factors were insignificant (|*slope*| ≤ 0.04).

**Figure 7:**
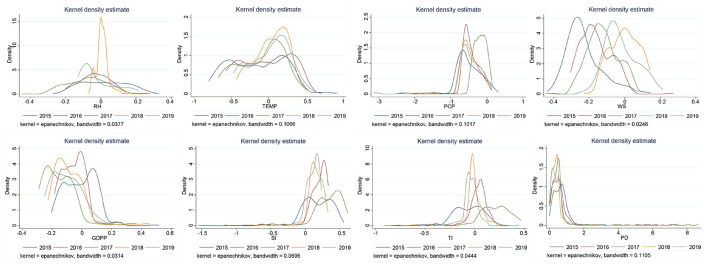
Kernel density distribution of each variable coefficient in different year.

The distribution characteristics of nuclear density of each coefficient are given in Figure 7. The change of left-biased peak distribution of RH was not obvious from 2015 to 2017, and it was concentrated in positive values in 2018, whereafter the largest density of coefficients of RH was distributed at –0.4 in 2019. The temperature coefficient shown a bimodal distribution, with a main peak of about 0.3, which indicates that the rising temperature will increase the concentration of PM_2.5_ in most cities. The coefficient of PCP was left-distributed during 2015~2019, but the peak has shifted significantly to the right in 2019, indicating that the negative driving ability was weakening. The coefficient of WS showed a multi-peak distribution from 2015 to 2019, except that the peak distribution was negative in 2018. Among the four socioeconomic factors, the GDPP coefficient showed a multi-peak distribution from 2015 to 2019. The main peak was promotion effect in 2015, and then turned negative. The coefficient of SI and TI showed a multi-peak distribution from 2015 to 2019, compared with the TI, the coefficient of SI showed a right distribution, and the coefficient of TI showed a double distribution, which was close to zero, indicating that the contribution of TI to PM_2.5_ is smaller than that of SI. The coefficients of PD had almost no negative values during 2015~2019, and had a multi-peak distribution.

Generally, among the natural factors selected in this study, except that temperature had obvious positive and negative driving effects on PM_2.5_ concentrations, the driving effects of PCP, WS and RH were mainly negative. In socioeconomic factors, GDPP and PM_2.5_ concentrations was two-way driving, PD, SI and TI had significant positive driving effects on PM_2.5_. The order of driving capability was PD >PCP >TEMP >SI >WS >TI >GDPP >RH. The coefficient of PD decreased most obvious in the whole study period (*slope*=–0.07), and the coefficients of SI and GDPP were decreased slightly, yet the coefficients of WS and TEMP showed an upward trend (0.06, 0.04; Supplementary Table S3). Using the global multiple regression model, a similar conclusion was reached, namely PD (positive) and PCP (negative) were the first two drivers of PM_2.5_ concentrations (*p* < 0.001; Supplementary Table S4).

## 4. Discussion

This research presented that the PM_2.5_ concentrations in different areas decreased with varying degrees, with an average decrease of 3.58 μg m^−3^ a^−1^. The series actions of energy-saving, emission-reduction and clean air proposed by the government in recent years have received some achievement. Central and East China with the higher mean PM_2.5_ concentrations have higher population densities, developed industries, intensive human activities and particulate matter emissions. The PM_2.5_ concentrations in North and central China hugely dropped may because that the developed industrial, agriculture and intensive human activities was controlled by the above actions.

Many previous studies have concluded that the severely polluted areas in China by PM_2.5_ were located in Beijing, Tianjin and Hebei and the surrounding areas (35, 42, 43). Some studies have also shown that Xinjiang has high concentrations of PM_2.5_ in China (44–47). North China was not the region with the highest PM_2.5_ concentrations in this study because it included Inner Mongolia and other areas with relatively low PM_2.5_ concentrations. The difference of these results may be due to the spatial scale. In addition, the lack of data in parts of Xinjiang also has some impact on the overall results of the country.

We demonstrated that the capacity of driving factors was PD >PCP >TEMP >SI >WS >TI >GDPP >RH. PD was the positive main driving factor, indicating that the increase of population density will lead to the rise of PM_2.5_ concentrations. The higher population density is frequently accompanied by high emissions from household activities (e.g., cooking, heating and smoking) and local transportation (48, 49). Besides, the traffic congestion caused by population agglomeration is unfavorable for the complete combustion of motor fuel (50). In addition, the region with higher population density is often accompanied by dense buildings, which is not conducive to the diffusion of PM_2.5_. The rational layout and design of urban buildings could promote the dispersion of pollutants and improve air quality (51), but the diffusion capacity of PM_2.5_ is rarely specified in urban building design. Exposure risk of toxic pollutants in densely populated areas is higher than that in sparsely populated areas (46). Therefore,we should pay attention to the effect of population density on PM_2.5_ concentrations.

PCP and RH were the negative main driving factors in this study, possibly because that they can enhance airborne PM_2.5_ condensation and deposition, thus reducing PM_2.5_ concentrations (52). SI had a positive driving effect on PM_2.5_ in most areas. It is well known that SI is dominated by heavy industries such as machinery, chemicals, and energy, and that it is the primary source of pollutants in the atmosphere. We found that the driving coefficient of the TI (0.11) was obvious small than that of the SI (0.22), which indicated that the TI also had a positive driving effect on PM_2.5_, but its driving capacity was equivalent to half of that of the SI. The nonlinear relationship occurred between GDPP and PM_2.5_ concentrations, such as PM_2.5_ concentrations in developed eastern regions was being controlled by advances in science and technology, as well as the optimization of industrial structure. Similar research conclusion has previously been discovered (26). Therefore, to ensure economic development while controlling pollution, the government should formulate waste emission standards, strengthen supervision and law enforcement, gradually optimize the industry, transition from SI to TI, implement strict emission standards, and compel polluting industrial enterprises to improve production capacity. Furthermore, regional industrial development should take into account the carrying capacity of the local natural environment, particularly fragile areas like the northwest.

The driving capability of PD was descending in space from northwest to southeast, which was reverse with the spatial distribution of population density. This might be because the ecological environment in Northwest China was more fragile, the environmental carrying capacity was lower, the available land was limited, and the industrial and agricultural activities were more concentrated. This study discovered that precipitation driving capability was significantly stronger in the north than that in the south, probably because the abundant rainfall in the south and the PM_2.5_ condensed has reached the threshold. Therefore, the precipitation appears to be more important for PM_2.5_ deposition in the north of China compared to the south China, due to the little rainfall, the dry climate, and the lower vegetation cover. Similar findings have been found in previous studies, which are subject to the law of diminishing marginal effect (53, 54). The result in this study is generally consistent with the that of previous studies (55).

In this study, the region of positive driving of temperature was mainly distributed in the north China and the Qinghai-Tibet Plateau with the lower annual-average temperature. The possible reason was that the annual-average temperature in these regions was relatively lower, and the increase in temperature on the flow of the atmosphere was not enough to make PM_2.5_ diffusion, instead, promoting the flow of dry surface particulate matter. On the contrary, even though the temperature was high in the southern region, the diffusion ability was enhanced, but the small surface dust is conducive to lower the PM_2.5_ concentrations. The driving capability of WS was stronger in the north than that in the south (Figure 4), it may be caused by flat terrain in the north which provides better diffusion conditions for atmospheric pollutants, and the increase in wind speed is more conducive to the diffusion of PM_2.5_, thereby improving regional air pollution (56). It is worth noting that WS has a stronger positive driving effect on PM_2.5_ concentrations in central and western Inner Mongolia, Xinjiang, Shaanxi, Gansu, and northeastern Sichuan. Due to these regions located on the Loess Plateau or the edge of the desert, the soil is relatively loose. When the wind speed reaches a certain level, it will also roll up loose dust on the ground, resulting in an increase in the concentrations of PM_2.5_ in the downwind area (22). The relationship of GDPP and PM_2.5_ concentrations were negative correlation in the central and eastern regions of China, especially in the Bohai Bay economic zone. While there was a weak positive correlation in the central and western regions, indicating that pollution may decrease as per capita GDP increases (57).

The two regions with the largest decrease in PM_2.5_ concentrations were North and Central China (*slope* = -3.99 μg m^−3^ a^−1^, *slope* = -3.41 μg m^−3^ a^−1^) (Figures 1, 2). Central China was the region with the highest mean PM_2.5_ concentrations in this study, and many studies have shown that Beijing-Tianjin-Hebei in North China has always been a high-value area of PM_2.5_ in China (53, 54). The two regions are located in mid-eastern region of China and are more developed in industry and agriculture. There are slight differences (relative humidity stabilized, temperature increased slightly, and precipitation and wind speed slightly decreased) in trends of four natural factors in North China. The trend of GDPP was increased, the trends of SI and PD decreased significantly, and the trend of TI was no significant. The main negative driving factors (PCP, RH, WS) and positive driving factors (PD, SI) showed a trend of decreasing in two regions, which indicated that the decline reason of PM_2.5_ concentrations might be due to the capability weakening of the positive driving factors (PD, SI). However, trends of TEMP and WS in Central China were opposite (weakly increased in North China and weakly reduced in Central China) that in North China, and the other factors were the similar (Supplementary Figure S4), but the driving directions of TEMP and WS were different in these regions, indicating that the causes of PM_2.5_ concentrations decrease in Central and North China were similarly. This result further shows that in the case of constant or even adverse natural factors, a series of emission reduction measures introduced by the state after 2013, such as increasing green area, limiting vehicles, industrial emission purification, coal gasification in heating facilities, and industrial transformation, can alleviate or even cover up the impact of population density increase on the increase of PM_2.5_ concentrations.

Overall, this study found that the annual-average values of the main negative driving factors (PCP and RH) showed a downward trend (-42.78mm·a^−1^, –0.39%· a^−1^), and the trends of WS and TEMP did not change significantly (0.01m·s^−1^· a^−1^) (Supplementary Figure S4), indicating that natural factors were not particularly favorable for driving the decrease of PM_2.5_ concentrations. Among the socioeconomic factors, except Northeast China, the trend of PD in other regions was rise, the trend of SI was decline significantly (-1.21%· a^−1^), while the trend of TI was increase (0.78%· a^−1^) (Supplementary Table S2). However, the positive main driving factor (PD) showed an upward trend, but the driving force of PD showed a significantly downward trend. The shift trend of industrial structure was from the secondary industry to the tertiary industry (the driving capacity of the secondary industry was higher that of the tertiary industry). It further demonstrated that the main reason for the decrease of PM_2.5_ concentrations may be the weakening of the driving ability of positive driving factor (PD) and the transfer from secondary industry to tertiary industry.

## 5. Conclusions

We comprehensively analyzed the spatial-temporal characteristic of PM_2.5_ and investigated the factors influencing PM_2.5_ concentrations by natural and socioeconomic factors in China. The results showed that 1) The mean PM_2.5_ concentrations was 37.55 ± 15.62 μg m^−3^ during 2015-2019, the decreasing trend of PM_2.5_ concentrations was 3.58 μg m^−3^ a^−1^, a decrease of 26.49% in 2019 compared to 2015, The regions with higher concentrations were mainly distributed in North China and South China, which were also the regions with the greatest decline in 5 years. 2) The capability of driving factors was PD >PCP >TEMP >SI >WS >TI >GDPP >RH, and the driving capability of socioeconomic factors on PM_2.5_ was slightly higher than that of natural factors. The strongest positive and negative driving factors were population density and precipitation, respectively. 3) North China and Central China were the two regions with the largest decreases in PM_2.5_ in the country from 2015 to 2019. The decrease in PM_2.5_ concentrations is primarily due to the implementation of a series of energy-saving and emission-reduction control measures after the Action Plan, such as clean air action and the adjustment of industrial structures by secondary and tertiary industries, which effectively offsets the impact of rising population density on PM_2.5_ concentrations.

The analysis above revealed that we should reduce PM_2.5_ concentration by improving socio-economic factors rather than waiting for natural factors to change. The industrial structure should be actively regulated and gradually changed from secondary to tertiary industry under the condition of ensuring stable economic growth, which is an important measure to ensure the socioeconomic effect while reducing PM_2.5_ concentrations. In the future, we must formulate a reasonable population policy so that population growth can be adapted to regional development, especially ecologically sensitive areas. In addition to we must consider environmental carrying capacity in urban planning and construction, balance population distribution, and other factors. Government departments should continue to develop and implement energy conservation and emission reduction measures in China, particularly densely populated areas, achieving win-win between economic development and environmental management.

## Data availability statement

The original contributions presented in the study are included in the article/Supplementary material, further inquiries can be directed to the corresponding author.

## Author contributions

YS: conceptualization, methodology, writing—original draft, and writing—review and editing. QC: methodology, formal analysis, and writing—review and editing. SY: data curation, methodology, writing—original draft, and writing—review and editing. PW: methodology, investigation, writing—review and editing, and supervision. BW: investigation and writing—review and editing. SZ: writing—review and editing. All authors contributed to the article and approved the submitted version.

## Funding

This work was supported by the National Natural Science Foundation of China (42167013).

## Conflict of interest

The authors declare that the research was conducted in the absence of any commercial or financial relationships that could be construed as a potential conflict of interest.

## Publisher's note

All claims expressed in this article are solely those of the authors and do not necessarily represent those of their affiliated organizations, or those of the publisher, the editors and the reviewers. Any product that may be evaluated in this article, or claim that may be made by its manufacturer, is not guaranteed or endorsed by the publisher.
