# Supplementary material for: Spatial-temporal heterogeneity and driving factors of PM2.5 in China: A natural and socioeconomic perspective
Source: Front Public Health. 2022 Nov 17;10:1051116. doi: 10.3389/fpubh.2022.1051116 (PMC9713317; doi:10.3389/fpubh.2022.1051116)
Supplement: Supplementary file 1 [file Data_Sheet_1.docx]

**Figure.S1** The classification of seven geographical regions in China


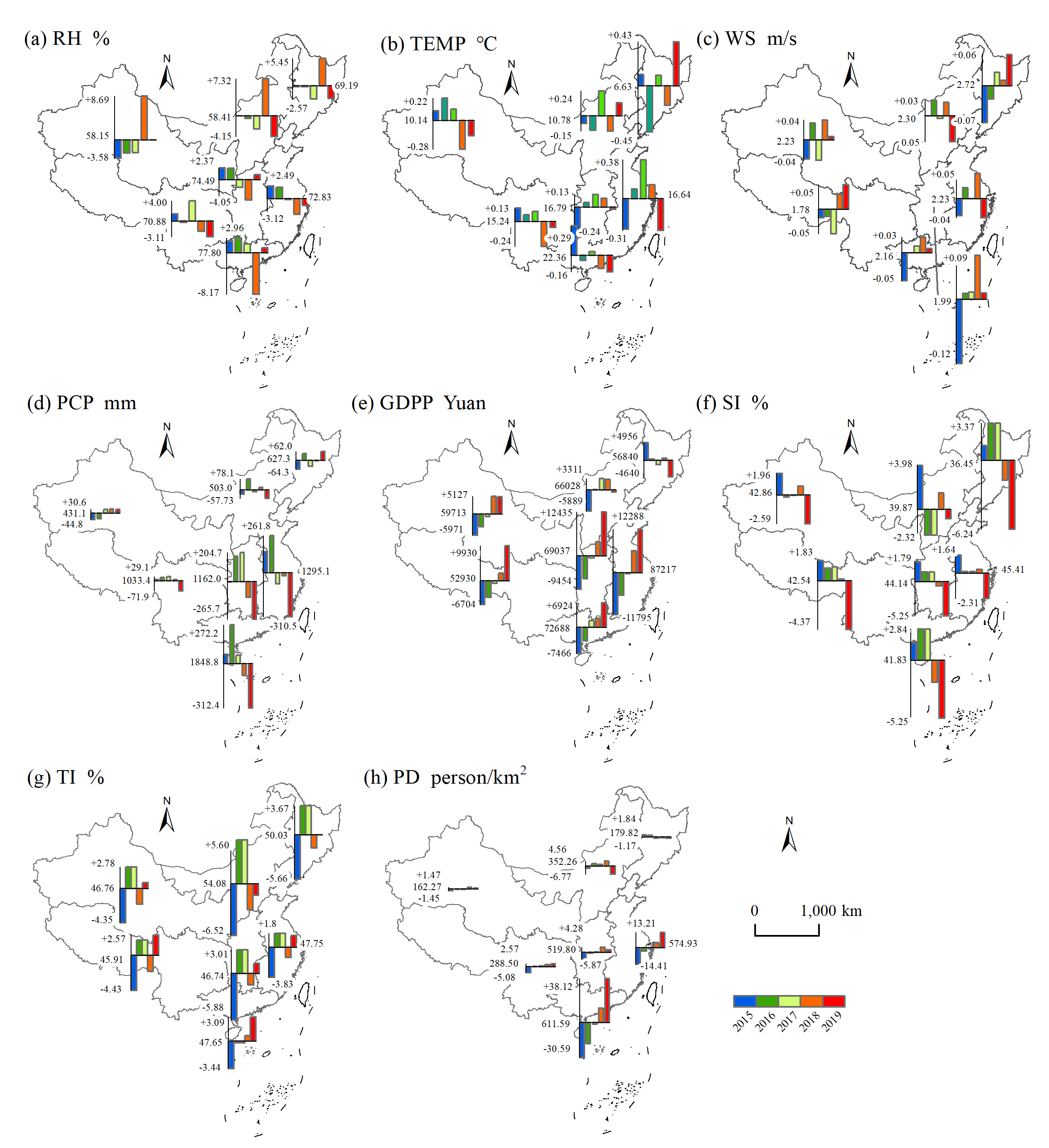
 **Figure. S2** Spatial distribution of natural and socioeconomic factors distance from 2015 to 2019 (a) Relative humidity, (b) Temperature, (c) Wind speed, (d) Precipitation, (e) per capita GDP, (f) Secondary industry share, (g) Tertiary Industry share, and (h) Population density


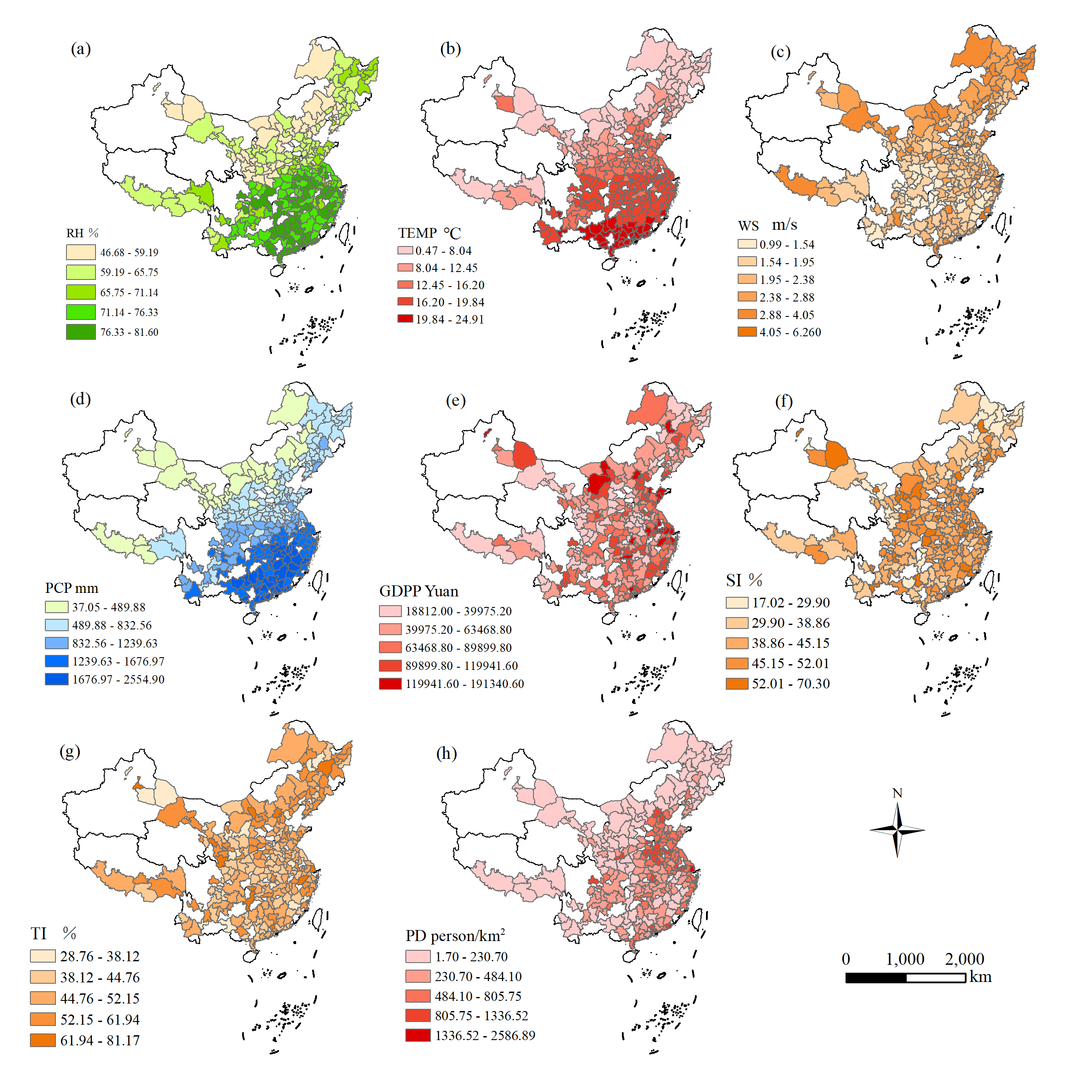
 **Figure.S3** Overall spatial distribution of natural and socioeconomic factors (average 2015 to 2019) (a) Relative humidity, (b) Temperature, (c) Wind speed, (d) Precipitation, (e) per capita GDP, (f) Secondary industry share, (g) Tertiary Industry share, and (h) Population density


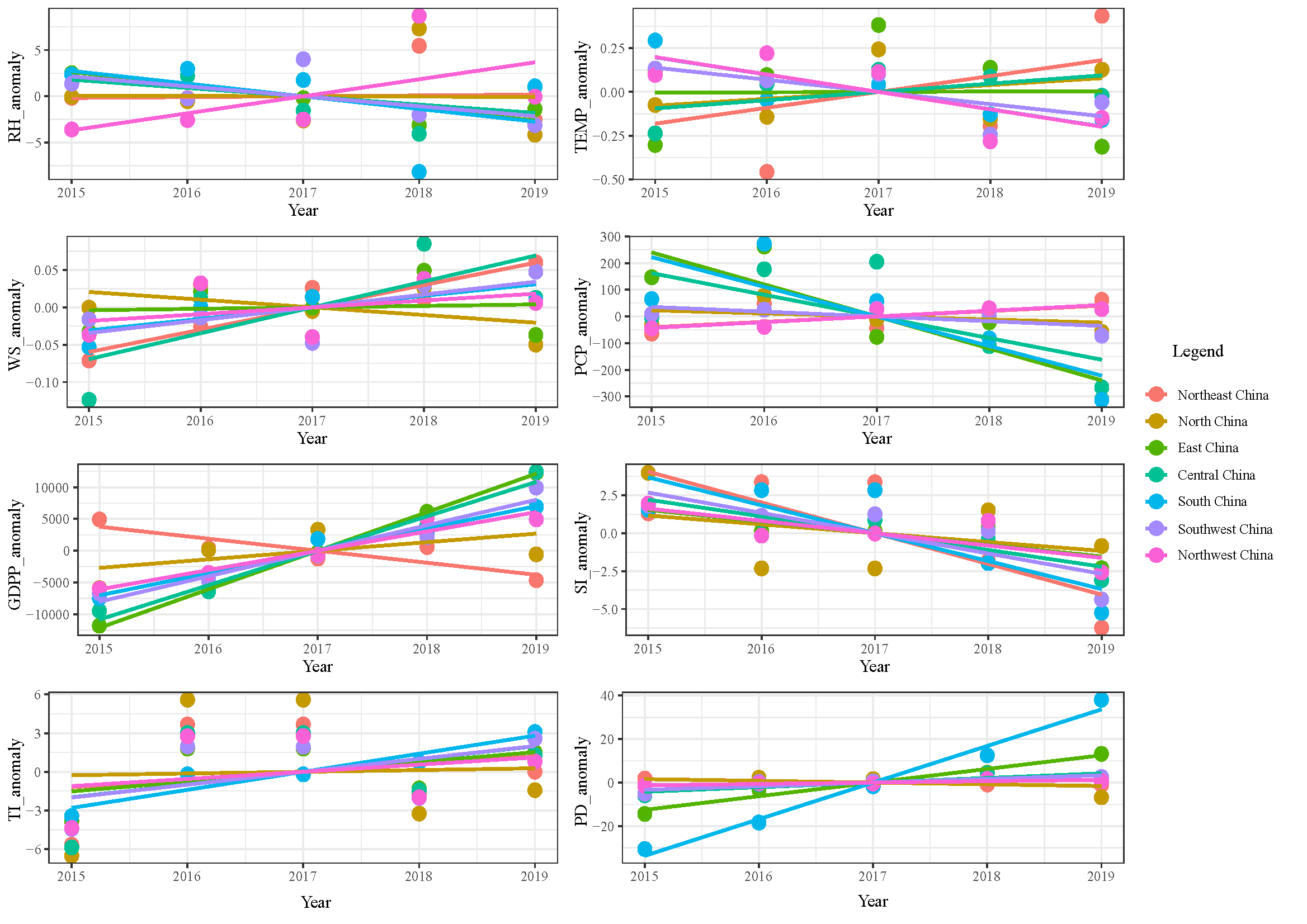


**Figure.S4** Trends in annual distance level of each factor from 2015 to 2019(line denotes the linear trend
