# Supplementary material for: Spatial-temporal heterogeneity and driving factors of PM2.5 in China: A natural and socioeconomic perspective
Source: Front Public Health. 2022 Nov 17;10:1051116. doi: 10.3389/fpubh.2022.1051116 (PMC9713317; doi:10.3389/fpubh.2022.1051116)
Supplement: Supplementary file 2 [file Data_Sheet_2.docx]

Table S1 Seven geographical subareas of Mainland China.

| Code | Geographical subarea | Provinces and municipalities |
| --- | --- | --- |
| Ⅰ | Northeast China | Liaoning, Jilin, and Heilongjiang |
| Ⅱ | North China | Beijing, Tianjin, Hebei, Shanxi and Inner Mongolia |
| Ⅲ | East China | Jiangsu, Zhejiang, Shanghai, Anhui, Fujian, Shandong and Jiangxi |
| Ⅳ | Central China | Hunan, Hubei and Henan |
| Ⅴ | South China | Guangxi, Hainan and Guangdong |
| Ⅵ | Southwest China | Sichuan, Yunnan, Guizhou, Chongqing and Tibet |
| Ⅶ | Northwest China | Gansu, Qinghai, Ningxia, Shaanxi and Xinjiang |

Table S2 Trends in natural and socioeconomic factors from 2015 to 2019 (slope)

| FQ | RH | TEMP | WS | PCP | GDPP | SI | TI | PD |  |
| --- | --- | --- | --- | --- | --- | --- | --- | --- | --- |
| Northeast | 0.09 | 0.09 | 0.03 | 20.21 | -1891.80 | -2.03 | 0.60 | -0.81 |  |
| North China | -0.04 | 0.04 | -0.01 | -11.46 | 1348.03 | -0.58 | 0.13 | -0.80 |  |
| East China | -1.31 | 0.00 | 0.00 | -119.79 | 6065.60 | -0.77 | 0.76 | 6.27 |  |
| Central China | | -0.90 | 0.05 | 0.03 | -81.05 | 5416.48 | -1.10 | 0.99 | 2.12 |
| South China | -1.37 | -0.10 | 0.02 | -110.95 | 3512.71 | -1.84 | 1.40 | 16.83 |  |
| Southwest | -1.07 | -0.07 | 0.02 | -17.84 | 4009.60 | -1.34 | 1.00 | 1.68 |  |
| Northwest | 1.83 | -0.10 | 0.01 | 21.39 | 3043.05 | -0.81 | 0.56 | 0.63 |  |
| Whole | -0.39 | -0.01 | 0.01 | -42.78 | 3071.95 | -1.21 | 0.78 | 3.70 |  |

Table S3 Characteristics of changes in regression coefficients of each factor

| Regions | | | factors | | 2015 | | | 2016 | 2017 | | 2018 | 2019 | slope | | average | | max | | min |
| --- | --- | --- | --- | --- | --- | --- | --- | --- | --- | --- | --- | --- | --- | --- | --- | --- | --- | --- | --- |
| Northeast China | | | RH | | -0.12 | | | -0.1 | -0.07 | | -0.04 | -0.07 | 0.02 | | -0.08 | | 0.75 | | -0.77 |
|  |  |  | TEMP | | 0.21 | | | 0.33 | 0.13 | | 0.21 | 0.16 | -0.02 | | 0.21 | |  |  |  |
|  |  |  | WS | | -0.28 | | | -0.25 | -0.13 | | -0.06 | -0.05 | 0.06 | | -0.15 | |  |  |  |
|  |  |  | PCP | | -0.77 | | | -0.54 | -0.26 | | -0.27 | -0.26 | 0.13 | | -0.42 | |  |  |  |
|  |  |  | GDPP | | -0.16 | | | -0.08 | -0.13 | | -0.15 | -0.18 | -0.01 | | -0.14 | |  |  |  |
|  |  |  | SI | | 0.38 | | | 0.16 | 0.24 | | 0.16 | 0.18 | -0.04 | | 0.22 | |  |  |  |
|  |  |  | TI | | 0.12 | | | 0.02 | 0.1 | | 0.02 | 0.02 | -0.02 | | 0.06 | |  |  |  |
|  |  |  | PD | | 0.75 | | | 0.56 | 0.57 | | 0.48 | 0.61 | -0.04 | | 0.6 | |  |  |  |
| North China | | | RH | | 0 | | | -0.09 | -0.15 | | 0 | -0.1 | -0.01 | | -0.07 | | 0.91 | | -0.76 |
|  |  |  | TEMP | | 0.27 | | | 0.36 | 0.29 | | 0.32 | 0.36 | 0.01 | | 0.32 | |  |  |  |
|  |  |  | WS | | -0.13 | | | -0.07 | -0.08 | | 0.04 | 0.08 | 0.05 | | -0.03 | |  |  |  |
|  |  |  | PCP | | -0.76 | | | -0.64 | -0.37 | | -0.62 | -0.6 | 0.03 | | -0.6 | |  |  |  |
|  |  |  | GDPP | | -0.11 | | | -0.15 | -0.24 | | -0.2 | -0.22 | -0.03 | | -0.18 | |  |  |  |
|  |  |  | SI | | 0.29 | | | 0.26 | 0.45 | | 0.28 | 0.19 | -0.02 | | 0.29 | |  |  |  |
|  |  |  | TI | | 0.02 | | | 0.09 | 0.3 | | 0.11 | 0.02 | 0 | | 0.11 | |  |  |  |
|  |  |  | PD | | 0.91 | | | 0.75 | 0.59 | | 0.5 | 0.58 | -0.09 | | 0.67 | |  |  |  |
| East China | | | RH | | -0.04 | | | -0.07 | -0.02 | | 0 | -0.08 | 0 | | -0.04 | | 0.44 | | -0.6 |
|  |  |  | TEMP | | -0.2 | | | -0.07 | -0.25 | | -0.03 | -0.04 | 0.04 | | -0.12 | |  |  |  |
|  |  |  | WS | | -0.28 | | | -0.19 | -0.21 | | -0.07 | -0.07 | 0.05 | | -0.17 | |  |  |  |
|  |  |  | PCP | | -0.59 | | | -0.54 | -0.25 | | -0.57 | -0.6 | 0 | | -0.51 | |  |  |  |
|  |  |  | GDPP | | -0.05 | | | -0.1 | -0.22 | | -0.13 | -0.14 | -0.02 | | -0.13 | |  |  |  |
|  |  |  | SI | | 0.32 | | | 0.27 | 0.44 | | 0.2 | 0.14 | -0.04 | | 0.27 | |  |  |  |
|  |  |  | TI | | 0.03 | | | 0.07 | 0.26 | | 0 | -0.02 | -0.02 | | 0.07 | |  |  |  |
|  |  |  | PD | | 0.39 | | | 0.32 | 0.32 | | 0.26 | 0.27 | -0.03 | | 0.31 | |  |  |  |
| Central China | | | RH | | 0.17 | | | 0.05 | -0.09 | | 0.03 | -0.01 | -0.04 | | 0.03 | | 0.53 | | -0.59 |
|  |  |  | TEMP | | -0.35 | | | -0.19 | -0.24 | | 0.01 | -0.04 | 0.08 | | -0.16 | |  |  |  |
|  |  |  | WS | | -0.23 | | | -0.14 | -0.1 | | 0.06 | 0.01 | 0.07 | | -0.08 | |  |  |  |
|  |  |  | PCP | | -0.5 | | | -0.49 | -0.17 | | -0.59 | -0.57 | -0.02 | | -0.47 | |  |  |  |
|  |  |  | GDPP | | 0.04 | | | -0.05 | -0.21 | | -0.09 | -0.09 | -0.03 | | -0.08 | |  |  |  |
|  |  |  | SI | | 0.12 | | | 0.24 | 0.47 | | 0.15 | 0.09 | -0.02 | | 0.21 | |  |  |  |
|  |  |  | TI | | -0.12 | | | 0.08 | 0.32 | | -0.01 | -0.07 | 0 | | 0.04 | |  |  |  |
|  |  |  | PD | | 0.53 | | | 0.42 | 0.34 | | 0.26 | 0.28 | -0.06 | | 0.37 | |  |  |  |
| South China | | | RH | | 0.11 | | | -0.05 | 0.08 | | 0.02 | 0.1 | 0.01 | | 0.05 | | 0.19 | | -0.63 |
|  |  |  | TEMP | | -0.63 | | | -0.51 | -0.49 | | -0.3 | -0.39 | 0.07 | | -0.46 | |  |  |  |
|  |  |  | WS | | -0.24 | | | -0.18 | -0.13 | | -0.01 | -0.07 | 0.05 | | -0.13 | |  |  |  |
|  |  |  | PCP | | -0.11 | | | -0.16 | 0.02 | | -0.16 | -0.13 | 0 | | -0.11 | |  |  |  |
|  |  |  | GDPP | | 0.08 | | | 0.01 | -0.08 | | -0.02 | 0 | -0.02 | | 0 | |  |  |  |
|  |  |  | SI | | -0.02 | | | 0.08 | 0.19 | | 0.07 | 0.02 | 0.01 | | 0.07 | |  |  |  |
|  |  |  | TI | | -0.15 | | | -0.02 | 0.09 | | -0.02 | -0.07 | 0.02 | | -0.03 | |  |  |  |
|  |  |  | PD | | 0.19 | | | 0.11 | 0.07 | | 0.05 | 0.08 | -0.03 | | 0.1 | |  |  |  |
| Southwest China | | | RH | | 0.04 | | | 0.06 | -0.14 | | 0.07 | 0.14 | 0.02 | | 0.03 | | 0.94 | | -0.32 |
|  |  |  | TEMP | | -0.32 | | | -0.19 | -0.06 | | -0.01 | -0.05 | 0.07 | | -0.13 | |  |  |  |
|  |  |  | WS | | -0.3 | | | -0.2 | -0.11 | | 0.05 | -0.07 | 0.07 | | -0.13 | |  |  |  |
|  |  |  | PCP | | -0.08 | | | -0.19 | -0.05 | | -0.22 | -0.23 | -0.03 | | -0.15 | |  |  |  |
|  |  |  | GDPP | | 0.1 | | | 0.04 | 0 | | 0.08 | 0.1 | 0 | | 0.06 | |  |  |  |
|  |  |  | SI | | -0.04 | | | 0.05 | 0.15 | | -0.02 | -0.06 | -0.01 | | 0.02 | |  |  |  |
|  |  |  | TI | | -0.27 | | | -0.09 | 0.02 | | -0.14 | -0.21 | 0.01 | | -0.14 | |  |  |  |
|  |  |  | PD | | 0.94 | | | 0.78 | 0.61 | | 0.49 | 0.47 | -0.12 | | 0.66 | |  |  |  |
| Northwest China | | | RH | | 0.03 | | | 0 | -0.25 | | 0 | -0.05 | -0.02 | | -0.05 | | 1.7 | | -0.77 |
|  |  |  | TEMP | | 0.06 | | | 0.11 | 0.15 | | 0.17 | 0.23 | 0.04 | | 0.14 | |  |  |  |
|  |  |  | WS | | -0.06 | | | -0.04 | 0 | | 0.07 | 0.09 | 0.04 | | 0.01 | |  |  |  |
|  |  |  | PCP | | -0.63 | | | -0.58 | -0.32 | | -0.7 | -0.77 | -0.04 | | -0.6 | |  |  |  |
|  |  |  | GDPP | | 0.05 | | | -0.01 | -0.08 | | -0.05 | -0.09 | -0.03 | | -0.04 | |  |  |  |
|  |  |  | SI | | -0.03 | | | 0.08 | 0.23 | | 0.07 | 0.1 | 0.02 | | 0.09 | |  |  |  |
|  |  |  | TI | | -0.15 | | | 0.01 | 0.15 | | -0.01 | -0.01 | 0.03 | | 0 | |  |  |  |
|  |  |  | PD | | 1.62 | | | 1.7 | 1.57 | | 1.22 | 1.15 | -0.14 | | 1.45 | |  |  |  |
| Whole region | | | RH | | 0.02 | | | -0.03 | -0.08 | | 0.01 | -0.02 | 0 | | -0.02 | | 0.71 | | -0.5 |
|  |  |  | TEMP | | -0.15 | | | -0.04 | -0.1 | | 0.04 | 0.02 | 0.04 | | -0.04 | |  |  |  |
|  |  |  | WS | | -0.23 | | | -0.16 | -0.12 | | 0 | -0.02 | 0.06 | | -0.11 | |  |  |  |
|  |  |  | PCP | | -0.5 | | | -0.46 | -0.2 | | -0.47 | -0.47 | 0.01 | | -0.42 | |  |  |  |
|  |  |  | GDPP | | -0.01 | | | -0.05 | -0.15 | | -0.09 | -0.1 | -0.02 | | -0.08 | |  |  |  |
|  |  |  | SI | | 0.17 | | | 0.18 | 0.33 | | 0.14 | 0.1 | -0.02 | | 0.18 | |  |  |  |
|  |  |  | TI | | -0.06 | | | 0.03 | 0.19 | | -0.01 | -0.05 | 0 | | 0.02 | |  |  |  |
|  |  |  | PD | | 0.71 | | | 0.61 | 0.55 | | 0.44 | 0.46 | -0.07 | | 0.55 | |  |  |  |
| Table S4 The results of the global multiple regression model | | | | | | | | | | | | | | | | |  | | |
| Model | | | Beta  (Unstandardized) | | | standard error | | | Beta  (standardized) | | | | t | | p | | Factors order | | |
|  | factors | | 44.247 | | 3.521 | | | |  | | | | 12.566 | | .000 | |  | | |
|  | RH | | -.077 | | .046 | | | | -.053 | | | | -1.658 | | .098 | | 6 | | |
|  | TEMP | | -.071 | | .129 | | | | -.024 | | | | -.546 | | .585 | | 8 | | |
|  | WS | | -2.555 | | .648 | | | | -.109 | | | | -3.945 | | .000 | | 5 | | |
|  | PCP | | -.008 | | .001 | | | | -.291 | | | | -7.598 | | .000 | | 2 | | |
|  | GDPP | | -6.369E-5 | | .000 | | | | -.148 | | | | -5.386 | | .000 | | 4 | | |
|  | SI | | .364 | | .041 | | | | .235 | | | | 8.861 | | .000 | | 3 | | |
|  | TI | | .006 | | .004 | | | | .035 | | | | 1.437 | | .151 | | 7 | | |
|  | PD | | .018 | | .001 | | | | .410 | | | | 13.839 | | .000 | | 1 | | |

Note:R=0.491, F=49.784, p<0.001;PD>PCP>SI>GDPP>WS>RH>TI>TEMP.
